# Benzoyl­methyl 4-chloro­benzoate

**DOI:** 10.1107/S1600536807066469

**Published:** 2008-01-23

**Authors:** Yi Jin, Jian-Nan Guo, Kan Lin, Guo Tang, Yu-Fen Zhao

**Affiliations:** a Department of Chemistry, The Key Laboratory for Chemical Biology of Fujian Province, College of Chemistry and Chemical Engineering, Xiamen University, Xiamen 361005, People’s Republic of China

## Abstract

The asymmetric unit of the title compound, C_15_H_11_ClO_3_, contains three mol­ecules, *A*, *B*, and *C*. Mol­ecules *A* and *B* are aligned edge-to-face, whereas mol­ecules *B* and *C* are aligned almost parallel to each other. The crystal structure displays C—H⋯π and π–π [centroid–centroid distances of 3.960 (4), 3.971 (4) and 3.971 (4) for mol­ecules *A*, *B* and *C*, respectively] parallel-displaced inter­actions, and C—H⋯O hydrogen bonds.

## Related literature

For background literature, see: Kelly & Howard (1932 ▶). For the synthesis, see Hendrickson & Kandall (1970 ▶). For bond-length data, see: Allen *et al.* (1987 ▶).
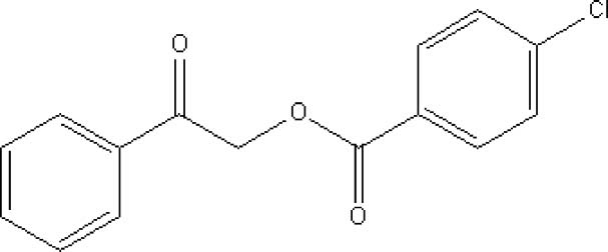


## Experimental

### 

#### Crystal data

C_15_H_11_ClO_3_
*M* *_r_* = 274.69
Monoclinic, 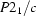

*a* = 14.7634 (6) Å
*b* = 16.4509 (6) Å
*c* = 15.8214 (5) Å
β = 92.105 (4)°
*V* = 3840.0 (2) Å^3^
*Z* = 12
Mo *K*α radiation
μ = 0.30 mm^−1^
*T* = 293 (2) K
0.18 × 0.15 × 0.10 mm

#### Data collection

Bruker APEX area-detector diffractometer
Absorption correction: multi-scan (*SADABS*; Bruker, 2001 ▶) *T*_min_ = 0.948, *T*_max_ = 0.971
18809 measured reflections
6647 independent reflections
3093 reflections with *I* > 2σ(*I*)
*R*_int_ = 0.061

#### Refinement

*R*[*F* ^2^ > 2σ(*F* ^2^)] = 0.037
*wR*(*F* ^2^) = 0.071
*S* = 0.79
6647 reflections
514 parameters
H-atom parameters constrained
Δρ_max_ = 0.16 e Å^−3^
Δρ_min_ = −0.18 e Å^−3^

### 

Data collection: *SMART* (Bruker, 2001 ▶); cell refinement: *SAINT* (Bruker, 2001 ▶); data reduction: *SAINT* (Bruker, 2001 ▶); program(s) used to solve structure: *SHELXS97* (Sheldrick, 1997 ▶); program(s) used to refine structure: *SHELXL97* (Sheldrick, 1997 ▶); molecular graphics: *ORTEP-3* (Farrugia, 1997 ▶); software used to prepare material for publication: *SHELXL97*

## Supplementary Material

Crystal structure: contains datablocks I, global. DOI: 10.1107/S1600536807066469/ng2408sup1.cif

Structure factors: contains datablocks I. DOI: 10.1107/S1600536807066469/ng2408Isup2.hkl

Additional supplementary materials:  crystallographic information; 3D view; checkCIF report

## Figures and Tables

**Table 1:** Hydrogen-bond geometry (Å, °)

*D*—H⋯*A*	*D*—H	H⋯*A*	*D*⋯*A*	*D*—H⋯*A*
C8*A*—H8*AB*⋯O3*B*	0.97	2.55	3.322 (3)	137
C8*C*—H8*CB*⋯O3*A*^i^	0.97	2.55	3.212 (3)	126
C11*A*—H11*A*⋯*Cg*1*B*	0.93	3.46	4.036 (4)	123
C11*B*—H11*B*⋯*Cg*1*A*	0.93	3.10	3.917 (4)	137

Symmetry code: (i) 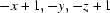. *Cg*1*A* and *Cg*1*B* are the centroids of the C1*A–*C6*A* and C1*B–*C6*B* benzene rings, respectively.

**Fig. 1.**
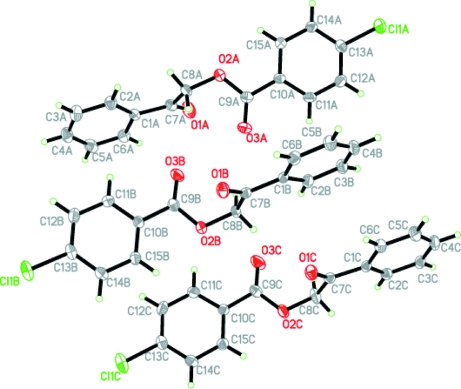
The asymmetric unit of (I), drawn with 50% probability displacement ellipsoids. H atoms are drawn as spheres of arbitrary radius.

**Fig. 2.**
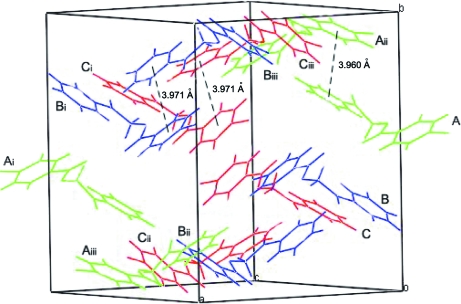
The unit cell of (I). The same subscript means the molecules form one asymmetric unit and the symmetry-equivalent molecules have the same color.

**Fig. 3.**
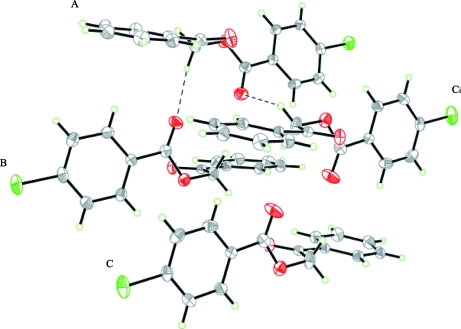
The asymmetric unit of molecule (I) which is formed by three molecules A, B, and Ci—symmetry-equivalent of C in the next asymmetric unit. Hydrogen bonds are indicated by dashed lines.

## supplementary crystallographic information

### Comment

An X-ray crystal structure determination of the molecule of the title compound, 2-oxo-2-phenylethyl 4-chlorobenzoate, (I) (Scheme. 1), was carried out to determine its conformation and the results are presented here. The bond lengths and angles are within their normal ranges (Allen *et al.*, 1987).

The unit cell has four identical asymmetric units which contain three molecules of (I) each (Fig. 2). In each asymmetric unit, the benzene rings of molecules B and C stack in a parallel-displaced structure, while the benzene rings of molecules A and B form a displaced T-shaped structure (Fig. 1). The two benzene rings within molecule A make a dihedral angle of 61.68 (6)°, while the two benzene rings within molecules B and C make almost identical dihedral angles of 77.66 (6)° and 77.29 (6)° respectively.

Hydrogen bonds C—H···O which are found not only between molecules A and B in the asymmetric unit but also between molecule A and the symmetry- equivalent Ci in the next asymmetric unit (Fig. 3). These C—H···O interactions stabilize the formation of asymmetric units as well as of the whole unit cell (Table 2).

Meanwhile, π—π interactions in stacked, slipped benzene rings from two symmetry-equivalent molecules in adjacent asymmetric units also provide stability for the crystal structure. The distance between *Cg*1 A and *Cg*2 A^ii^ is 3.960 (4) Å and the angles between the line through the centroids of these two benzene rings and the normal through *Cg*1 A is 26.6 (1)° and through *Cg*2 A^ii^ is 25.0 (1)°. The corresponding values for benzene rings C1B/C6B and C10B/C15B^iii^ are 3.971 (4) Å, 26.4 (1)° and 24.9 (1)°. For C1C/C6C and C10C/C15C^iii^ they are 3.971 (4) Å, 18.1 (1)° and 33.5 (1)°.*Cg*1X and *Cg*2X are the centroids of C1/C6 and C10/C15 benzene rings in molecule *X* (*X*=A, B, C) respectively [Symmetry code: (ii) *x*, -*y* - 1/2, *z* + 1/2; (iii) *x*, -*y* + 1/2, *z* - 1/2] (Fig. 2). The packing is further stabilized by weak C—H···π interactions(Table 1).The distances and angles correspond to their caculated ranges.

### Experimental

Triethylamine (0.84 ml, 6 mmol) was slowly dropped into 2-bromo-1-phenylethanone (796 mg, 4 mmol) and 4-chlorobenzoic acid (630 mg, 4 mmol) dissolved in freshly distilled tetrahydrofuran (10 ml) at rt under nitrogen and stirred overnight. The precipitate was collected at the pump and washed with ethyl acetate. The filtrate and washings were combined and back-washed successively with 1/3 of the volume each of 10% citric acid, 10% sodium bicarbonate, and water and then dried. Solvent was distilled off *in vacuo* and the residue recrystallized repeatedly from ethyl acetate-petroleum ether giving 1.085 g (98%) as colourless needles, m.p.128°C. (Hendrickson & Kandall, 1970).

### Refinement

The hydrogen atoms were generated geometrically (C—H = 0.93, 0.98, 0.97 or 0.96Å for phenyl, tertiary, methylene or methyl H atoms respectively, and N—H = 0.86 Å) and were included in the refinement in the riding model approximation. The displacement parameters of methyl H atoms were set to 1.5 times *U*_eq_ of the equivalent isotropic displacement parameters of their parent atoms, while those of other H atoms were set to 1.2 times.

### Crystal data

**Table:**

C_15_H_11_ClO_3_	*F*_000_ = 1704
*M**_r_* = 274.69	*D*_x_ = 1.425 Mg m^−^^3^
Monoclinic, *P*2_1_/*c*	Melting point: 128 K
Hall symbol: -P 2ybc	Mo *K*α radiation λ = 0.71073 Å
*a* = 14.7634 (6) Å	Cell parameters from 3122 reflections
*b* = 16.4509 (6) Å	θ = 2.5–32.7º
*c* = 15.8214 (5) Å	µ = 0.30 mm^−^^1^
β = 92.105 (4)º	*T* = 293 (2) K
*V* = 3840.0 (2) Å^3^	Needle, colorless
*Z* = 12	0.18 × 0.15 × 0.10 mm

### Data collection

**Table:**

Bruker APEX area-detector diffractometer	6647 independent reflections
Radiation source: fine-focus sealed tube	3093 reflections with *I* > 2σ(*I*)
Monochromator: graphite	*R*_int_ = 0.061
*T* = 293(2) K	θ_max_ = 25.0º
φ and ω scans	θ_min_ = 2.5º
Absorption correction: multi-scan(SADABS; Bruker, 2001)	*h* = −17→17
*T*_min_ = 0.948, *T*_max_ = 0.971	*k* = −19→19
18809 measured reflections	*l* = −18→18

### Refinement

**Table:**

Refinement on *F*^2^	Secondary atom site location: difference Fourier map
Least-squares matrix: full	Hydrogen site location: inferred from neighbouring sites
*R*[*F*^2^ > 2σ(*F*^2^)] = 0.037	H-atom parameters constrained
*wR*(*F*^2^) = 0.071	*w* = 1/[σ^2^(*F*_o_^2^) + (0.0229*P*)^2^] where *P* = (*F*_o_^2^ + 2*F*_c_^2^)/3
*S* = 0.79	(Δ/σ)_max_ = 0.001
6647 reflections	Δρ_max_ = 0.16 e Å^−^^3^
514 parameters	Δρ_min_ = −0.18 e Å^−^^3^
Primary atom site location: structure-invariant direct methods	Extinction correction: none

### Special details

**Table:**

Geometry. All e.s.d.'s (except the e.s.d. in the dihedral angle between two l.s. planes) are estimated using the full covariance matrix. The cell e.s.d.'s are taken into account individually in the estimation of e.s.d.'s in distances, angles and torsion angles; correlations between e.s.d.'s in cell parameters are only used when they are defined by crystal symmetry. An approximate (isotropic) treatment of cell e.s.d.'s is used for estimating e.s.d.'s involving l.s. planes.
Refinement. Refinement of *F*^2^ against ALL reflections. The weighted *R*-factor *wR* and goodness of fit *S* are based on *F*^2^, conventional *R*-factors *R* are based on *F*, with *F* set to zero for negative *F*^2^. The threshold expression of *F*^2^ > σ(*F*^2^) is used only for calculating *R*-factors(gt) *etc*. and is not relevant to the choice of reflections for refinement. *R*-factors based on *F*^2^ are statistically about twice as large as those based on *F*, and *R*- factors based on ALL data will be even larger.

### Fractional atomic coordinates and isotropic or equivalent isotropic displacement parameters (Å^2^)

**Table:**

	*x*	*y*	*z*	*U*_iso_*/*U*_eq_	
Cl1A	0.03772 (5)	−0.08212 (4)	0.11747 (4)	0.0556 (2)	
Cl1B	0.26822 (6)	0.11374 (5)	1.12967 (4)	0.0633 (2)	
Cl1C	0.61726 (6)	0.10917 (5)	1.00742 (4)	0.0683 (3)	
O2A	0.03489 (12)	−0.10047 (10)	0.53569 (9)	0.0424 (5)	
O1B	0.19554 (12)	0.18589 (10)	0.65265 (10)	0.0436 (5)	
O2B	0.32721 (12)	0.09438 (10)	0.71717 (9)	0.0402 (5)	
O1C	0.50058 (12)	0.18749 (10)	0.51078 (10)	0.0450 (5)	
O3A	0.16842 (13)	−0.03651 (11)	0.52509 (10)	0.0499 (5)	
O3B	0.21291 (13)	0.00485 (11)	0.72245 (10)	0.0488 (5)	
O2C	0.63757 (12)	0.11032 (11)	0.59116 (10)	0.0447 (5)	
O1A	0.14699 (14)	−0.20470 (10)	0.62048 (10)	0.0561 (6)	
C8A	0.04329 (18)	−0.09541 (15)	0.62601 (13)	0.0385 (7)	
H8AA	−0.0167	−0.0971	0.6491	0.046*	
H8AB	0.0707	−0.0437	0.6417	0.046*	
C7C	0.55633 (18)	0.15783 (14)	0.46475 (15)	0.0323 (6)	
C1C	0.55205 (18)	0.17052 (14)	0.37173 (14)	0.0311 (6)	
C10A	0.08523 (17)	−0.07507 (14)	0.39938 (15)	0.0309 (6)	
C7B	0.24177 (18)	0.15393 (14)	0.60005 (14)	0.0308 (6)	
C1A	0.09642 (18)	−0.17310 (14)	0.75762 (14)	0.0324 (6)	
O3C	0.53403 (16)	0.01186 (11)	0.60006 (11)	0.0650 (6)	
C8B	0.31582 (17)	0.09562 (15)	0.62639 (13)	0.0373 (7)	
H8BA	0.3006	0.0415	0.6060	0.045*	
H8BB	0.3721	0.1120	0.6016	0.045*	
C10B	0.27073 (18)	0.06419 (14)	0.85060 (14)	0.0309 (6)	
C3C	0.6090 (2)	0.15411 (15)	0.23220 (16)	0.0403 (7)	
H3CA	0.6525	0.1340	0.1967	0.048*	
C10C	0.59559 (18)	0.07204 (15)	0.72688 (14)	0.0318 (6)	
C7A	0.09950 (18)	−0.16285 (15)	0.66412 (15)	0.0366 (7)	
C12A	0.13340 (19)	−0.04395 (15)	0.25981 (16)	0.0415 (7)	
H12A	0.1753	−0.0213	0.2242	0.050*	
C3B	0.2621 (2)	0.15140 (16)	0.36190 (16)	0.0459 (8)	
H3BA	0.2972	0.1270	0.3214	0.055*	
C2C	0.61796 (18)	0.14046 (14)	0.31906 (15)	0.0357 (7)	
H2CA	0.6673	0.1116	0.3416	0.043*	
C1B	0.22630 (17)	0.17034 (14)	0.50769 (14)	0.0282 (6)	
C9B	0.26507 (19)	0.04978 (16)	0.75790 (15)	0.0357 (7)	
C13B	0.2710 (2)	0.09279 (16)	1.02189 (15)	0.0417 (7)	
C4A	0.1008 (2)	−0.19577 (16)	0.93128 (17)	0.0508 (8)	
H4AA	0.1021	−0.2034	0.9896	0.061*	
C12B	0.2066 (2)	0.04234 (16)	0.98578 (16)	0.0470 (8)	
H12B	0.1633	0.0183	1.0189	0.056*	
C6A	0.16530 (19)	−0.21753 (15)	0.79763 (16)	0.0424 (7)	
H6AA	0.2105	−0.2402	0.7657	0.051*	
C12C	0.5516 (2)	0.03615 (17)	0.86616 (17)	0.0470 (8)	
H12C	0.5170	0.0049	0.9018	0.056*	
C11A	0.14801 (18)	−0.04141 (14)	0.34670 (15)	0.0385 (7)	
H11A	0.2001	−0.0170	0.3697	0.046*	
C3A	0.0322 (2)	−0.15192 (16)	0.89305 (17)	0.0503 (8)	
H3AA	−0.0130	−0.1297	0.9254	0.060*	
C2B	0.27901 (18)	0.13470 (14)	0.44678 (14)	0.0390 (7)	
H2BA	0.3258	0.0996	0.4631	0.047*	
C9C	0.5839 (2)	0.05997 (17)	0.63423 (16)	0.0391 (7)	
C6B	0.15735 (18)	0.22224 (15)	0.48194 (16)	0.0404 (7)	
H6BA	0.1213	0.2461	0.5221	0.048*	
C9A	0.1030 (2)	−0.06842 (16)	0.49227 (16)	0.0374 (7)	
C11B	0.20653 (18)	0.02740 (15)	0.89963 (15)	0.0412 (7)	
H11B	0.1635	−0.0072	0.8747	0.049*	
C5C	0.4711 (2)	0.22617 (15)	0.25039 (16)	0.0481 (8)	
H5CA	0.4218	0.2549	0.2275	0.058*	
C5B	0.1412 (2)	0.23927 (16)	0.39721 (16)	0.0514 (8)	
H5BA	0.0948	0.2748	0.3806	0.062*	
C2A	0.02969 (19)	−0.14037 (15)	0.80593 (15)	0.0425 (7)	
H2AA	−0.0171	−0.1105	0.7802	0.051*	
C13A	0.05596 (19)	−0.08045 (15)	0.22680 (14)	0.0379 (7)	
C13C	0.61072 (19)	0.09358 (16)	0.89888 (15)	0.0395 (7)	
C6C	0.47918 (19)	0.21258 (15)	0.33635 (16)	0.0401 (7)	
H6CA	0.4347	0.2321	0.3712	0.048*	
C15B	0.33538 (18)	0.11417 (15)	0.88873 (14)	0.0371 (7)	
H15A	0.3791	0.1383	0.8561	0.045*	
C8C	0.63088 (19)	0.10529 (17)	0.50074 (14)	0.0498 (8)	
H8CA	0.6197	0.0493	0.4841	0.060*	
H8CB	0.6879	0.1220	0.4777	0.060*	
C14C	0.66371 (19)	0.13928 (15)	0.84783 (15)	0.0408 (7)	
H14A	0.7046	0.1769	0.8709	0.049*	
C14A	−0.00688 (18)	−0.11492 (14)	0.27799 (14)	0.0375 (7)	
H14B	−0.0586	−0.1397	0.2547	0.045*	
C15A	0.00808 (17)	−0.11202 (14)	0.36445 (14)	0.0351 (6)	
H15B	−0.0339	−0.1351	0.3997	0.042*	
C4C	0.5369 (2)	0.19671 (15)	0.19875 (16)	0.0481 (8)	
H4CA	0.5321	0.2060	0.1408	0.058*	
C4B	0.1938 (2)	0.20361 (16)	0.33722 (17)	0.0507 (8)	
H4BA	0.1830	0.2150	0.2802	0.061*	
C15C	0.65510 (19)	0.12822 (15)	0.76153 (15)	0.0381 (7)	
H15C	0.6902	0.1593	0.7261	0.046*	
C14B	0.33586 (19)	0.12884 (15)	0.97478 (14)	0.0405 (7)	
H14C	0.3794	0.1626	1.0002	0.049*	
C11C	0.54441 (19)	0.02561 (15)	0.77951 (16)	0.0436 (7)	
H11C	0.5047	−0.0131	0.7566	0.052*	
C5A	0.1678 (2)	−0.22867 (16)	0.88403 (16)	0.0498 (8)	
H5AA	0.2146	−0.2583	0.9102	0.060*	

### Atomic displacement parameters (Å^2^)

**Table:**

	*U*^11^	*U*^22^	*U*^33^	*U*^12^	*U*^13^	*U*^23^
Cl1A	0.0629 (5)	0.0707 (5)	0.0334 (4)	−0.0070 (4)	0.0027 (4)	−0.0032 (4)
Cl1B	0.0917 (7)	0.0693 (5)	0.0292 (3)	0.0110 (5)	0.0060 (4)	−0.0045 (4)
Cl1C	0.0927 (7)	0.0810 (6)	0.0312 (3)	0.0232 (5)	0.0041 (4)	−0.0038 (4)
O2A	0.0411 (12)	0.0550 (12)	0.0309 (9)	−0.0050 (10)	−0.0012 (9)	0.0031 (8)
O1B	0.0521 (12)	0.0455 (11)	0.0337 (9)	0.0056 (10)	0.0100 (10)	−0.0054 (9)
O2B	0.0402 (11)	0.0540 (12)	0.0264 (9)	−0.0049 (10)	−0.0011 (8)	0.0029 (8)
O1C	0.0497 (13)	0.0514 (12)	0.0344 (10)	0.0079 (10)	0.0094 (10)	−0.0046 (9)
O3A	0.0404 (12)	0.0647 (13)	0.0440 (11)	−0.0114 (11)	−0.0084 (10)	−0.0061 (10)
O3B	0.0611 (14)	0.0502 (12)	0.0344 (10)	−0.0144 (11)	−0.0086 (10)	−0.0042 (9)
O2C	0.0411 (12)	0.0656 (12)	0.0269 (9)	0.0029 (10)	−0.0038 (9)	0.0029 (9)
O1A	0.0757 (15)	0.0533 (12)	0.0391 (10)	0.0234 (11)	0.0011 (11)	−0.0099 (9)
C8A	0.0394 (17)	0.0472 (17)	0.0287 (13)	0.0009 (15)	−0.0038 (13)	−0.0007 (13)
C7C	0.0325 (17)	0.0310 (15)	0.0336 (14)	−0.0046 (13)	0.0040 (14)	−0.0043 (12)
C1C	0.0373 (17)	0.0277 (15)	0.0287 (14)	−0.0042 (13)	0.0053 (14)	−0.0016 (12)
C10A	0.0257 (15)	0.0293 (14)	0.0375 (14)	0.0051 (13)	−0.0024 (13)	0.0016 (12)
C7B	0.0334 (16)	0.0278 (14)	0.0314 (14)	−0.0074 (13)	0.0030 (13)	−0.0004 (12)
C1A	0.0377 (17)	0.0267 (14)	0.0326 (14)	0.0002 (13)	−0.0017 (14)	0.0009 (12)
O3C	0.0987 (18)	0.0496 (13)	0.0450 (12)	−0.0208 (13)	−0.0213 (12)	−0.0034 (10)
C8B	0.0364 (16)	0.0540 (18)	0.0214 (13)	−0.0004 (15)	0.0004 (12)	0.0017 (12)
C10B	0.0350 (16)	0.0291 (15)	0.0283 (13)	0.0026 (13)	−0.0020 (13)	0.0025 (12)
C3C	0.054 (2)	0.0368 (16)	0.0307 (15)	−0.0088 (15)	0.0095 (15)	−0.0034 (13)
C10C	0.0322 (16)	0.0310 (15)	0.0321 (14)	0.0005 (13)	−0.0004 (13)	0.0004 (12)
C7A	0.0409 (18)	0.0323 (16)	0.0363 (15)	0.0005 (14)	−0.0044 (14)	−0.0045 (13)
C12A	0.0369 (18)	0.0436 (17)	0.0443 (17)	−0.0007 (14)	0.0064 (15)	0.0054 (14)
C3B	0.059 (2)	0.0492 (18)	0.0297 (15)	−0.0075 (17)	0.0031 (15)	−0.0003 (13)
C2C	0.0390 (17)	0.0309 (16)	0.0373 (15)	−0.0013 (13)	0.0019 (14)	0.0006 (12)
C1B	0.0305 (15)	0.0269 (14)	0.0275 (13)	−0.0050 (13)	0.0020 (13)	0.0022 (11)
C9B	0.0392 (18)	0.0354 (17)	0.0323 (15)	0.0047 (14)	−0.0011 (15)	0.0024 (13)
C13B	0.058 (2)	0.0411 (17)	0.0256 (13)	0.0114 (16)	0.0024 (15)	0.0006 (13)
C4A	0.076 (2)	0.0398 (18)	0.0366 (16)	−0.0080 (17)	0.0003 (18)	0.0053 (14)
C12B	0.051 (2)	0.0533 (19)	0.0371 (16)	−0.0015 (16)	0.0096 (15)	0.0073 (14)
C6A	0.0469 (19)	0.0384 (16)	0.0419 (16)	0.0031 (15)	0.0005 (15)	0.0017 (13)
C12C	0.0436 (19)	0.0537 (19)	0.0441 (17)	−0.0007 (16)	0.0084 (15)	0.0184 (15)
C11A	0.0258 (16)	0.0411 (16)	0.0481 (16)	−0.0021 (13)	−0.0058 (13)	0.0046 (14)
C3A	0.063 (2)	0.0428 (18)	0.0457 (17)	0.0089 (17)	0.0123 (17)	0.0036 (14)
C2B	0.0449 (18)	0.0398 (17)	0.0326 (14)	−0.0033 (14)	0.0066 (14)	0.0011 (12)
C9C	0.0426 (19)	0.0356 (17)	0.0384 (16)	0.0087 (15)	−0.0075 (15)	0.0016 (14)
C6B	0.0382 (17)	0.0396 (17)	0.0434 (16)	0.0024 (14)	0.0040 (14)	0.0064 (13)
C9A	0.0322 (17)	0.0385 (17)	0.0414 (16)	0.0012 (15)	−0.0026 (15)	−0.0008 (13)
C11B	0.0400 (18)	0.0454 (17)	0.0381 (15)	−0.0085 (15)	0.0006 (14)	0.0010 (13)
C5C	0.055 (2)	0.0457 (18)	0.0425 (16)	0.0073 (16)	−0.0039 (16)	0.0080 (14)
C5B	0.054 (2)	0.0512 (19)	0.0484 (17)	−0.0018 (16)	−0.0063 (16)	0.0168 (15)
C2A	0.0457 (19)	0.0434 (17)	0.0384 (15)	0.0069 (15)	0.0015 (14)	0.0049 (13)
C13A	0.0427 (18)	0.0385 (16)	0.0324 (14)	0.0063 (15)	0.0007 (14)	−0.0045 (13)
C13C	0.0446 (18)	0.0431 (18)	0.0309 (14)	0.0148 (15)	0.0026 (14)	0.0005 (13)
C6C	0.0440 (19)	0.0391 (16)	0.0373 (16)	0.0023 (14)	0.0027 (14)	0.0036 (13)
C15B	0.0392 (17)	0.0418 (16)	0.0305 (14)	0.0018 (15)	0.0032 (13)	0.0033 (13)
C8C	0.051 (2)	0.073 (2)	0.0256 (14)	0.0195 (17)	0.0028 (14)	0.0040 (14)
C14C	0.0473 (19)	0.0351 (17)	0.0395 (16)	0.0008 (14)	−0.0072 (15)	−0.0062 (13)
C14A	0.0362 (17)	0.0408 (16)	0.0357 (14)	−0.0067 (14)	0.0025 (13)	−0.0046 (13)
C15A	0.0331 (16)	0.0378 (15)	0.0343 (14)	−0.0023 (14)	0.0022 (13)	0.0013 (12)
C4C	0.070 (2)	0.0449 (18)	0.0295 (15)	−0.0065 (17)	0.0021 (17)	0.0055 (14)
C4B	0.069 (2)	0.0494 (19)	0.0327 (15)	−0.0193 (17)	−0.0060 (16)	0.0113 (14)
C15C	0.0439 (18)	0.0345 (16)	0.0361 (15)	−0.0050 (14)	0.0037 (14)	0.0005 (13)
C14B	0.0472 (19)	0.0392 (17)	0.0345 (15)	0.0007 (15)	−0.0062 (14)	−0.0011 (13)
C11C	0.0432 (19)	0.0444 (17)	0.0427 (16)	−0.0075 (15)	−0.0070 (14)	0.0061 (14)
C5A	0.057 (2)	0.0438 (18)	0.0473 (17)	0.0044 (16)	−0.0130 (16)	0.0116 (14)

### Geometric parameters (Å, °)

**Table:**

Cl1A—C13A	1.741 (2)	C5C—C6C	1.379 (3)
O1A—C7A	1.216 (2)	C6A—H6AA	0.9300
C1A—C2A	1.378 (3)	C6B—H6BA	0.9300
C1A—C6A	1.386 (3)	C6C—H6CA	0.9300
C1A—C7A	1.491 (3)	C7A—C8A	1.499 (3)
Cl1B—C13B	1.742 (2)	C7B—C8B	1.502 (3)
O1B—C7B	1.215 (2)	C7C—C8C	1.495 (4)
C1B—C2B	1.390 (3)	C8A—H8AA	0.9700
C1B—C6B	1.379 (3)	C8A—H8AB	0.9700
C1B—C7B	1.495 (3)	C8B—H8BA	0.9700
Cl1C—C13C	1.736 (2)	C8B—H8BB	0.9700
O1C—C7C	1.221 (2)	C8C—H8CA	0.9700
C1C—C2C	1.395 (3)	C8C—H8CB	0.9700
C1C—C6C	1.380 (3)	C9A—C10A	1.487 (3)
C1C—C7C	1.486 (3)	C9B—C10B	1.485 (3)
O2A—C8A	1.432 (3)	C9C—C10C	1.483 (3)
O2A—C9A	1.346 (3)	C10A—C11A	1.385 (3)
C2A—H2AA	0.9300	C10A—C15A	1.388 (3)
C2A—C3A	1.391 (3)	C10B—C11B	1.386 (3)
O2B—C8B	1.440 (3)	C10B—C15B	1.382 (3)
O2B—C9B	1.356 (3)	C10C—C11C	1.376 (3)
C2B—H2BA	0.9300	C10C—C15C	1.375 (3)
C2B—C3B	1.384 (3)	C11A—H11A	0.9300
O2C—C8C	1.433 (3)	C11A—C12A	1.384 (3)
O2C—C9C	1.349 (3)	C11B—H11B	0.9300
C2C—H2CA	0.9300	C11B—C12B	1.385 (3)
C2C—C3C	1.394 (3)	C11C—H11C	0.9300
O3A—C9A	1.200 (3)	C11C—C12C	1.382 (3)
C3A—H3AA	0.9300	C12A—H12A	0.9300
C3A—C4A	1.366 (4)	C12A—C13A	1.377 (4)
O3B—C9B	1.193 (3)	C12B—H12B	0.9300
C3B—H3BA	0.9300	C12B—C13B	1.370 (4)
C3B—C4B	1.370 (4)	C12C—H12C	0.9300
O3C—C9C	1.196 (3)	C12C—C13C	1.375 (4)
C3C—H3CA	0.9300	C13A—C14A	1.376 (3)
C3C—C4C	1.364 (4)	C13B—C14B	1.370 (3)
C4A—H4AA	0.9300	C13C—C14C	1.370 (3)
C4A—C5A	1.373 (3)	C14A—H14B	0.9300
C4B—H4BA	0.9300	C14A—C15A	1.378 (3)
C4B—C5B	1.379 (3)	C14B—H14C	0.9300
C4C—H4CA	0.9300	C14B—C15B	1.382 (3)
C4C—C5C	1.381 (3)	C14C—H14A	0.9300
C5A—H5AA	0.9300	C14C—C15C	1.379 (3)
C5A—C6A	1.378 (3)	C15A—H15B	0.9300
C5B—H5BA	0.9300	C15B—H15A	0.9300
C5B—C6B	1.382 (3)	C15C—H15C	0.9300
C5C—H5CA	0.9300		
			
Cl1A—C13A—C14A	119.7 (2)	C4C—C5C—C6C	119.3 (3)
Cl1A—C13A—C12A	118.7 (2)	C5A—C4A—H4AA	119.8
O1A—C7A—C1A	122.6 (2)	C5A—C6A—H6AA	119.5
O1A—C7A—C8A	120.9 (2)	C5B—C4B—H4BA	120.1
C1A—C2A—H2AA	119.9	C5B—C6B—H6BA	119.6
C1A—C2A—C3A	120.2 (3)	C5C—C4C—H4CA	119.8
C1A—C6A—H6AA	119.5	C5C—C6C—H6CA	119.4
C1A—C6A—C5A	121.0 (3)	C6A—C1A—C7A	117.7 (2)
C1A—C7A—C8A	116.5 (2)	C6A—C5A—H5AA	120.2
Cl1B—C13B—C14B	119.3 (2)	C6B—C1B—C7B	119.0 (2)
Cl1B—C13B—C12B	119.2 (2)	C6B—C5B—H5BA	120.0
O1B—C7B—C1B	121.4 (2)	C6C—C1C—C7C	118.5 (2)
O1B—C7B—C8B	120.5 (2)	C6C—C5C—H5CA	120.4
C1B—C2B—H2BA	119.9	C7A—C8A—H8AA	109.1
C1B—C2B—C3B	120.2 (3)	C7A—C8A—H8AB	109.1
C1B—C6B—H6BA	119.6	C7B—C8B—H8BA	109.7
C1B—C6B—C5B	120.8 (2)	C7B—C8B—H8BB	109.7
C1B—C7B—C8B	118.0 (2)	C7C—C8C—H8CA	109.3
Cl1C—C13C—C14C	119.4 (2)	C7C—C8C—H8CB	109.3
Cl1C—C13C—C12C	119.1 (2)	C8A—O2A—C9A	116.6 (2)
O1C—C7C—C1C	122.0 (2)	H8AA—C8A—H8AB	107.8
O1C—C7C—C8C	120.5 (2)	C8B—O2B—C9B	115.2 (2)
C1C—C2C—H2CA	120.4	H8BA—C8B—H8BB	108.2
C1C—C2C—C3C	119.3 (3)	C8C—O2C—C9C	116.7 (2)
C1C—C6C—H6CA	119.4	H8CA—C8C—H8CB	107.9
C1C—C6C—C5C	121.3 (2)	C9A—C10A—C11A	117.9 (3)
C1C—C7C—C8C	117.5 (2)	C9A—C10A—C15A	122.6 (2)
O2A—C8A—H8AA	109.1	C9B—C10B—C11B	117.9 (3)
O2A—C8A—H8AB	109.1	C9B—C10B—C15B	122.6 (2)
O2A—C8A—C7A	112.62 (19)	C9C—C10C—C11C	118.5 (3)
O2A—C9A—O3A	123.7 (2)	C9C—C10C—C15C	122.3 (2)
O2A—C9A—C10A	111.5 (3)	C10A—C11A—H11A	119.9
C2A—C1A—C6A	118.7 (2)	C10A—C11A—C12A	120.2 (3)
C2A—C1A—C7A	123.7 (3)	C10A—C15A—H15B	119.7
C2A—C3A—H3AA	119.9	C10A—C15A—C14A	120.6 (2)
C2A—C3A—C4A	120.2 (3)	C10B—C11B—H11B	120.1
O2B—C8B—H8BA	109.7	C10B—C11B—C12B	119.8 (3)
O2B—C8B—H8BB	109.7	C10B—C15B—H15A	119.6
O2B—C8B—C7B	110.05 (18)	C10B—C15B—C14B	120.8 (2)
O2B—C9B—O3B	123.3 (2)	C10C—C11C—H11C	119.8
O2B—C9B—C10B	111.6 (2)	C10C—C11C—C12C	120.4 (3)
C2B—C1B—C6B	118.8 (2)	C10C—C15C—H15C	119.4
C2B—C1B—C7B	122.1 (2)	C10C—C15C—C14C	121.2 (2)
C2B—C3B—H3BA	119.8	C11A—C10A—C15A	119.5 (2)
C2B—C3B—C4B	120.4 (3)	C11A—C12A—H12A	120.4
O2C—C8C—H8CA	109.3	C11A—C12A—C13A	119.1 (2)
O2C—C8C—H8CB	109.3	C11B—C10B—C15B	119.4 (2)
O2C—C8C—C7C	111.7 (2)	C11B—C12B—H12B	120.2
O2C—C9C—O3C	122.8 (2)	C11B—C12B—C13B	119.6 (2)
O2C—C9C—C10C	111.6 (3)	C11C—C10C—C15C	119.3 (2)
C2C—C1C—C6C	119.1 (2)	C11C—C12C—H12C	120.5
C2C—C1C—C7C	122.5 (3)	C11C—C12C—C13C	119.0 (2)
C2C—C3C—H3CA	119.7	C12A—C11A—H11A	119.9
C2C—C3C—C4C	120.6 (3)	C12A—C13A—C14A	121.6 (2)
O3A—C9A—C10A	124.7 (2)	C12B—C11B—H11B	120.1
C3A—C2A—H2AA	119.9	C12B—C13B—C14B	121.6 (2)
C3A—C4A—H4AA	119.8	C12C—C11C—H11C	119.8
C3A—C4A—C5A	120.3 (3)	C12C—C13C—C14C	121.6 (2)
O3B—C9B—C10B	125.1 (2)	C13A—C12A—H12A	120.4
C3B—C2B—H2BA	119.9	C13A—C14A—H14B	120.6
C3B—C4B—H4BA	120.1	C13A—C14A—C15A	118.9 (3)
C3B—C4B—C5B	119.8 (3)	C13B—C12B—H12B	120.2
O3C—C9C—C10C	125.7 (3)	C13B—C14B—H14C	120.6
C3C—C2C—H2CA	120.4	C13B—C14B—C15B	118.8 (3)
C3C—C4C—H4CA	119.8	C13C—C12C—H12C	120.5
C3C—C4C—C5C	120.5 (2)	C13C—C14C—H14A	120.7
C4A—C3A—H3AA	119.9	C13C—C14C—C15C	118.5 (3)
C4A—C5A—H5AA	120.2	C14A—C15A—H15B	119.7
C4A—C5A—C6A	119.6 (3)	C14B—C15B—H15A	119.6
C4B—C3B—H3BA	119.8	C14C—C15C—H15C	119.4
C4B—C5B—H5BA	120.0	C15A—C14A—H14B	120.6
C4B—C5B—C6B	120.0 (3)	C15B—C14B—H14C	120.6
C4C—C3C—H3CA	119.7	C15C—C14C—H14A	120.7
C4C—C5C—H5CA	120.4		
			
Cl1A—C13A—C12A—C11A	179.21 (19)	O3B—C9B—C10B—C15B	176.9 (3)
Cl1A—C13A—C14A—C15A	−179.1 (2)	C3B—C2B—C1B—C6B	−0.0 (4)
O1A—C7A—C8A—O2A	14.7 (3)	C3B—C2B—C1B—C7B	179.8 (2)
O1A—C7A—C1A—C2A	−164.7 (2)	C3B—C4B—C5B—C6B	−0.1 (4)
O1A—C7A—C1A—C6A	15.9 (4)	C8C—O2C—C9C—O3C	1.5 (4)
C1A—C2A—C3A—C4A	−0.0 (4)	O3C—C9C—C10C—C11C	−1.2 (4)
C1A—C6A—C5A—C4A	0.5 (4)	O3C—C9C—C10C—C15C	179.3 (3)
C1A—C7A—C8A—O2A	−167.6 (2)	C3C—C2C—C1C—C6C	0.5 (4)
Cl1B—C13B—C12B—C11B	177.9 (2)	C3C—C2C—C1C—C7C	179.4 (2)
Cl1B—C13B—C14B—C15B	−177.6 (2)	C3C—C4C—C5C—C6C	0.5 (4)
O1B—C7B—C8B—O2B	−7.1 (3)	C5A—C6A—C1A—C7A	179.1 (2)
O1B—C7B—C1B—C2B	179.5 (2)	C5B—C6B—C1B—C7B	179.7 (2)
O1B—C7B—C1B—C6B	−0.7 (4)	C5C—C6C—C1C—C7C	−179.8 (2)
C1B—C2B—C3B—C4B	0.5 (4)	C6A—C1A—C7A—C8A	−161.8 (2)
C1B—C6B—C5B—C4B	0.6 (4)	C6B—C1B—C7B—C8B	178.5 (2)
C1B—C7B—C8B—O2B	173.7 (2)	C6C—C1C—C7C—C8C	174.2 (2)
Cl1C—C13C—C12C—C11C	178.0 (2)	C7A—C8A—O2A—C9A	−83.8 (3)
Cl1C—C13C—C14C—C15C	−177.6 (2)	C7B—C8B—O2B—C9B	78.7 (2)
O1C—C7C—C8C—O2C	−12.2 (4)	C7C—C8C—O2C—C9C	86.1 (3)
O1C—C7C—C1C—C2C	176.6 (2)	C8A—O2A—C9A—C10A	−178.2 (2)
O1C—C7C—C1C—C6C	−4.5 (4)	C8B—O2B—C9B—C10B	−169.2 (2)
C1C—C2C—C3C—C4C	0.3 (4)	C8C—O2C—C9C—C10C	−178.8 (2)
C1C—C6C—C5C—C4C	0.4 (4)	C9A—C10A—C11A—C12A	−178.2 (2)
C1C—C7C—C8C—O2C	169.1 (2)	C9A—C10A—C15A—C14A	178.2 (2)
O2A—C9A—C10A—C11A	177.32 (19)	C9B—C10B—C11B—C12B	−176.9 (2)
O2A—C9A—C10A—C15A	−1.5 (3)	C9B—C10B—C15B—C14B	177.1 (2)
C2A—C1A—C6A—C5A	−0.4 (4)	C9C—C10C—C11C—C12C	−178.5 (3)
C2A—C1A—C7A—C8A	17.6 (4)	C9C—C10C—C15C—C14C	178.9 (3)
C2A—C3A—C4A—C5A	−0.1 (4)	C10A—C11A—C12A—C13A	0.2 (4)
O2B—C9B—C10B—C11B	175.1 (2)	C10A—C15A—C14A—C13A	0.1 (4)
O2B—C9B—C10B—C15B	−3.1 (3)	C10B—C11B—C12B—C13B	−0.7 (4)
C2B—C1B—C6B—C5B	−0.6 (4)	C10B—C15B—C14B—C13B	0.1 (4)
C2B—C1B—C7B—C8B	−1.2 (3)	C10C—C11C—C12C—C13C	0.1 (4)
C2B—C3B—C4B—C5B	−0.5 (4)	C10C—C15C—C14C—C13C	−0.8 (4)
O2C—C9C—C10C—C11C	179.2 (2)	C11A—C10A—C15A—C14A	−0.5 (4)
O2C—C9C—C10C—C15C	−0.3 (4)	C11A—C12A—C13A—C14A	−0.4 (4)
C2C—C1C—C6C—C5C	−0.8 (4)	C11B—C10B—C15B—C14B	−1.0 (4)
C2C—C1C—C7C—C8C	−4.8 (4)	C11B—C12B—C13B—C14B	0.2 (4)
C2C—C3C—C4C—C5C	−0.8 (4)	C11C—C10C—C15C—C14C	−0.6 (4)
O3A—C9A—O2A—C8A	0.2 (4)	C11C—C12C—C13C—C14C	−1.3 (4)
O3A—C9A—C10A—C11A	−0.7 (4)	C12A—C11A—C10A—C15A	0.6 (4)
O3A—C9A—C10A—C15A	−179.5 (2)	C12A—C13A—C14A—C15A	0.6 (4)
C3A—C2A—C1A—C6A	0.1 (4)	C12B—C11B—C10B—C15B	1.3 (4)
C3A—C2A—C1A—C7A	−179.3 (3)	C12B—C13B—C14B—C15B	0.6 (4)
C3A—C4A—C5A—C6A	−0.3 (4)	C12C—C11C—C10C—C15C	1.0 (4)
O3B—C9B—O2B—C8B	10.9 (3)	C12C—C13C—C14C—C15C	1.7 (4)
O3B—C9B—C10B—C11B	−4.9 (4)		

### Hydrogen-bond geometry (Å, °)

**Table:**

*D*—H···*A*	*D*—H	H···*A*	*D*···*A*	*D*—H···*A*
C8A—H8AB···O3B	0.97	2.55	3.322 (3)	137
C8C—H8CB···O3A^i^	0.97	2.55	3.212 (3)	126
C11A—H11A···Cg1B	0.93	3.46	4.036 (4)	123
C11B—H11B···Cg1A	0.93	3.10	3.917 (4)	137

Symmetry codes: (i) −*x*+1, −*y*, −*z*+1.
